# Clinical validation of video-based vital sign monitoring in the intensive care unit: a prospective cohort study: Clinical validation of video-based vital sign monitoring

**DOI:** 10.1007/s10877-026-01440-0

**Published:** 2026-05-13

**Authors:** Iris Cramer, Rik van Esch, Cindy Verstappen, Carla Kloeze, Jos Twisk, Sander Stuijk, Jan Bergmans, Svitlana Zinger, Ashley De Bie Dekker, Marcel van ’t Veer, R. Arthur Bouwman, Lukas Dekker, Leon Montenij

**Affiliations:** 1 Department of Electrical Engineering, Eindhoven University of Technology, Groene loper 3, 5612 AZ Eindhoven, The Netherlands; 2 Department of Anesthesiology, Catharina Hospital, Eindhoven, The Netherlands; 3 Department of Anesthesiology, Radboud University Medical Center, Nijmegen, The Netherlands; 4 Department of Intensive Care, Catharina Hospital, Eindhoven, The Netherlands; 5 Department of Cardiology, Catharina Hospital Eindhoven, Eindhoven, The Netherlands; 6 Department of Medical physics, Catharina Hospital, Eindhoven, The Netherlands; 7 Department of Epidemiology and Data science, Amsterdam University Medical Center, Amsterdam, The Netherlands; 8 Department of Intensive Care, Anna Hospital, Geldrop, The Netherlands

**Keywords:** Physiologic monitoring, Video monitoring, Heart rate, Respiratory rate, Critical care

## Abstract

**Purpose:**

Video-based monitoring technologies enable continuous and contactless monitoring of vital signs. This study evaluates the clinical concordance and determinants of performance of contactless video-based heart and respiratory rate monitoring compared with reference standards in a heterogeneous population of critically ill patients.

**Methods:**

In this prospective observational study, 35 intensive care unit patients were continuously monitored for 24 h. Video-based heart rate and respiratory rate were compared with the clinical reference standard. Agreement was assessed using Bland–Altman plots, intraclass correlation coefficients (ICC), and error grid analyses. Generalized estimating equations (GEE) identified factors affecting agreement.

**Results:**

For heart rate, bias was 2.1 bpm (limits − 33.6 to 37.7), with 81.9% within ± 5 bpm and 99.3% in error grid zones A/B. ICC was 0.43. For respiratory rate, bias was − 2.4 breaths/min (limits − 14.3 to 9.5), with 63.5% within ± 3 breaths and 87.5% in zones A/B. ICC was 0.41. High heart rate, atrial fibrillation, norepinephrine administration, and movement reduced agreement for heart rate; movement reduced agreement for respiratory rate.

**Conclusion:**

Video-based monitoring shows promise for detecting abnormal vital signs in critically ill patients, but improved robustness to motion is needed for reliable clinical implementation.

**Supplementary Information:**

The online version contains supplementary material available at https://doi.org/10.1007/s10877-026-01440-0.

## Introduction

 Video-based, contactless monitoring has emerged as a promising approach to enable continuous and unobtrusive surveillance of vital signs, particularly in lower-acuity clinical environments where mobility and comfort are priorities [1–3]. To meaningfully enhance patient safety, however, comprehensive evaluations are needed to determine whether such technology can reliably support clinical practice under diverse and high-stakes conditions.

Over recent years, technical feasibility studies have demonstrated that video-based measurement of heart and respiratory rates can provide accurate estimates in controlled or semi-controlled environments [1, 2]. These systems may ultimately support contextual monitoring, extending surveillance beyond vital signs alone in perioperative care [4]. Yet, most work to date has concentrated on algorithm development, with limited validation in the clinical scenarios where accuracy is most critical [5, 6]. Currently, video-data under conditions of physiological instability are lacking for further algorithm development and validation [7]. Addressing these uncertainties is essential to determine the clinical robustness and appropriate application of this technology across heterogeneous patient populations and situations.

Although contactless video-based monitoring is primarily intended for low-acuity care settings, evaluation in a high-acuity environment provides a rigorous testbed across a broad spectrum of pathophysiological conditions. The aim of this study is to assess clinical agreement between contactless video-based measurements of heart rate and respiratory rate and reference methods in critically ill patients. In addition, we explore patient-specific and physiological determinants of measurement agreement to inform safe implementation in lower-acuity settings.

## Methods

### Design and setting

A prospective, longitudinal study was performed among patients admitted to the intensive care unit (ICU) (Catharina hospital, Eindhoven, the Netherlands). The study was reviewed by the medical ethical committee of the MEC-U (Nieuwegein, the Netherlands, File no: W20.180), and the internal review boards of Catharina hospital Eindhoven. The study was registered at clinicaltrials.gov with the registry number: NCT06099327. Written informed consent from the patient or family (deferred consent) was obtained prior to starting the research procedures.

### Study population and procedures

Patients eligible for inclusion were adults (≥ 18 years) admitted to the ICU between August 2022 – August 2023. The following subgroups were defined: patients after elective cardiac surgery, patients admitted because of respiratory failure (defined as Pa/Fio2 ratio < 300) and hemodynamic instability (defined as need for vasopressors to maintain mean arterial pressure > 65 mmHg). No formal sample size calculation was made because there are no established guidelines in the literature for method comparison studies involving multiple sequential measurements per patient. Therefore, we aimed to include as many patients as was practically feasible within the study period, resulting in a sample size of 35. The focus of each recording was to capture approximately 24 h of monitoring per patient, allowing inclusion of patient-specific trends, either towards recovery or towards complications. This approach aims to provide a rich dataset covering a wide range of physiological states and clinical conditions. The resulting volume and diversity of data were considered sufficient to explore agreement patterns and influencing factors, consistent with previous repeated-measures method comparison studies [6]. Continuous video recording was started upon admission and lasted 24 h or until the patient was discharged. Patient characteristics such as admission reason, surgical category, age, sex, and Fitzpatrick skin type were collected. In addition, heart rhythm was classified as sinus rhythm, atrial fibrillation, or other [8]. The administration of continuous intravenous medication and all laboratory values obtained during the admission were also recorded. Patient movement was quantified based on facial pixel motion and categorized into three categories: no movement, slight movement and substantial movement [9]. The study population is the same as that included in our prior study [9], in which different cameras were used.

### Investigational setup

**Fig. 1:**
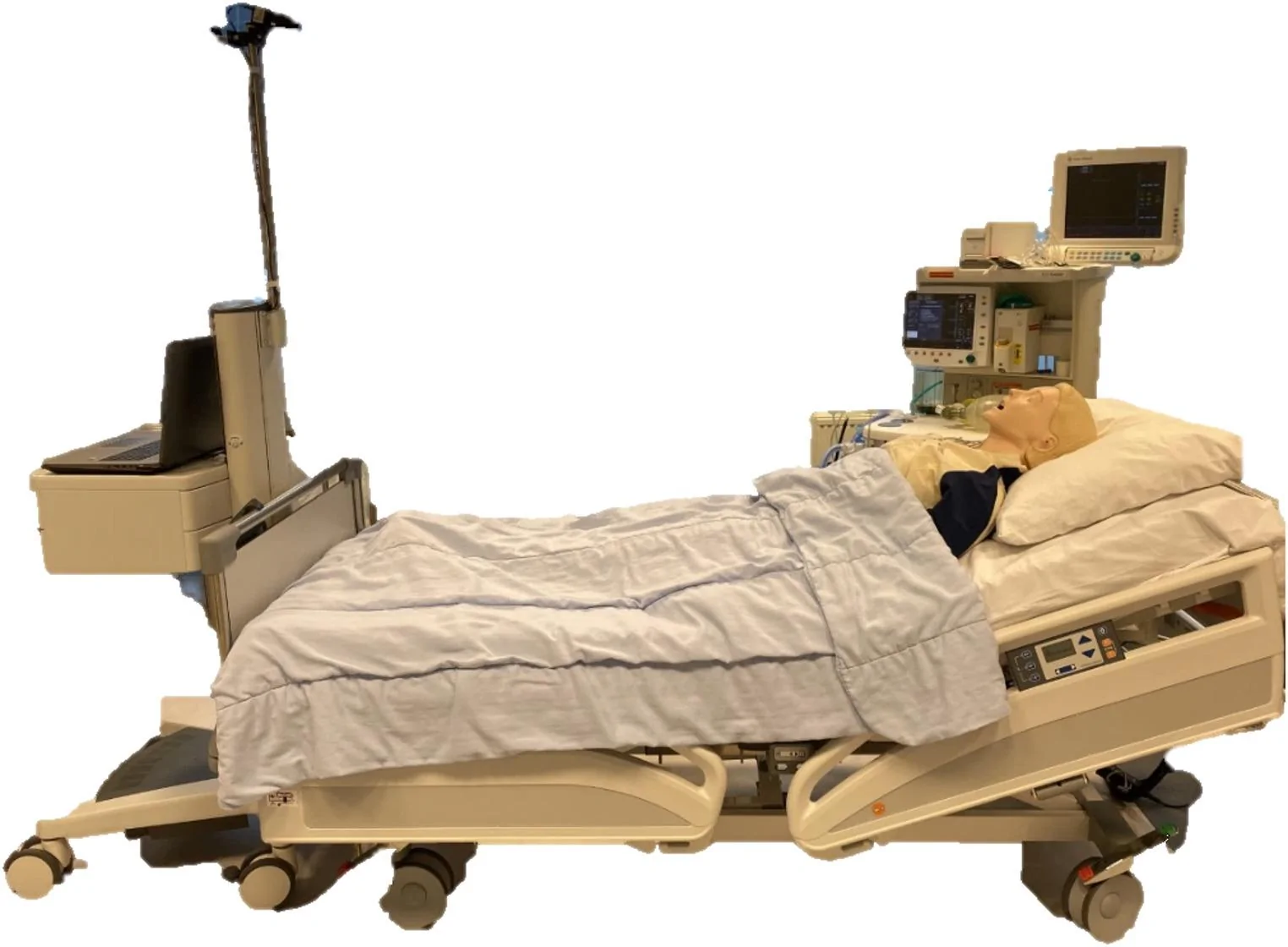
Investigational setup

Patients were monitored with a video camera that was mounted on a specifically developed medical-grade trolley (iTD Pro-cart) and positioned at the foot-end of the patients’ bed, approximately two meters from the patient’s face (Fig. 1). The videos were recorded using a single RGB camera (model 3860-C-HQ Rev. 2) with a frame rate of 32 Hz and a spatial resolution of 484 × 274 pixels (IDS Imaging Development Systems GmbH, Obersulm, Germany). The camera was equipped with fixed 8 mm focal length lenses (TAMRON M118FM08). The video acquisition was performed on a laptop (HP) with Visual Studio 2017 (Microsoft). A detailed description of the hardware setup, acquisition protocol, and processing pipeline has been reported previously [9, 10].

### Reference standard

Heart rate was derived from single-lead ECG and respiratory rate from impedance pneumography and airway pressure, as computed by a standard bedside patient monitoring system (Philips MP70/MX750). The reference measurements were exported via IXtrend and Data Warehouse Connect (Philips) using the monitor-calculated vital sign values.

### Data preprocessing

Post-processing of the video data was performed using MATLAB 2023b (MathWorks, USA) and is described in detail in previous work [9]. First, inadequate recordings, which comprised periods during which the patient was out of scope, or when the cameras were disabled to ensure patient privacy, were manually annotated and subsequently excluded from further analysis. The face was defined as the primary region of interest (ROI) for rPPG extraction. Common facial occlusions in the ICU, such as oxygen masks and nasogastric tubes, were not excluded from the analysis. Next, the rPPG waveform was retrieved by utilizing the Plane-Orthogonal-to-Skin (POS) method of Wang et al. [11]. For each patient, the entire video recording was segmented into consecutive 60-second analysis windows. Within each window, heart rate was estimated by identifying the dominant frequency peak in the corresponding Fourier spectrum of the rPPG signal [9]. The video-based respiratory rate signal was acquired by detection of respiratory-induced motion patterns [12]. The chest was defined as the primary region of interest. Periods during which the chest was not visible were manually annotated. All video-derived vital signs were synchronized with the bedside monitor reference measurements to ensure accurate temporal alignment. Reliable heart rate estimation from RGB imaging depends on sufficient ambient illumination, while low-light conditions require infrared acquisition. As only RGB-based measurements were used in this study, episodes with inadequate lighting were excluded based on illumination quality thresholds we previously developed and validated in our previous work [9]. Each 60-second window was processed by an atrial fibrillation detection algorithm to classify the heart rhythm [8].

### Statistical analysis

Data were either presented as means with standard deviations (SD), as medians with 25th and 75th percentiles, or as absolute numbers (with percentages).

Bland Altman plots were used to visualize and quantify the mean difference and 95% limits of agreement (LoA) between the video vital signs and reference vital signs [13]. To account for repeated measurements within individuals, we estimated the LoA using the Bland Altman method for repeated measures. A linear mixed-effects model was fitted with the measurement difference as the dependent variable and a random intercept for patient, allowing separation of between-patient and within-patient variance components. The total variance (sum of the between- and within-patient components) was used to derive the LoA. The harmonic mean of the number of paired observations per patient was applied to adjust for the unbalanced number of repeated measurements across patients, as recommended by Bland and Altman and subsequent methodological extensions [14]. Reliability was expressed as intra-class correlation (ICC) and determined using two-way mixed effects models [15, 16].

Next, an Error Grid analysis was conducted to evaluate the potential clinical consequences of measurement discrepancies for treatment decisions. As the Clarke Error Grid was originally developed and validated for glucose monitoring, its use for heart rate and respiratory rate in this study represents an adaptation to vital sign monitoring. The Error Grid was constructed based on clinically adapted thresholds derived from the Modified Early Warning Score (MEWS), as originally described by Subbe et al. [17], with categories consolidated to ensure sufficient observations per group and to maintain model stability [5, 6]. Zone A includes data points within ± 20% of the reference value or correct classification as bradycardia/bradypnea. Zone B contains points deviating by more than 20% from the reference but unlikely to lead to inappropriate treatment. Zone C includes measurements that could result in unnecessary treatment in patients with normal vital signs. Zone D reflects missed detection of bradycardia/bradypnea or tachycardia/tachypnoea, while Zone E indicates misclassification between opposite clinical states (e.g., bradycardia vs. tachycardia). The numerical thresholds used for classification were as follows: heart rate < 40 bpm (bradycardia), 40–100 bpm (normal), 100–130 bpm (elevated), and > 130 bpm (severely elevated); respiratory rate < 8 breaths/min (bradypnea), 8–20 breaths/min (normal), 20–25 breaths/min (elevated), and > 25 breaths/min (severely elevated). As MEWS thresholds may vary across implementations, and categories were adapted in this study, the resulting zone classification may be sensitive to the selected cut-off values, which should be considered when interpreting the Error Grid results.

Finally, multivariable logistic Generalized Estimating Equations (GEE) analyses were used to identify clinical factors associated with discrepancies between video-derived vital signs and the reference standard. For heart rate, discrepancy was defined as a difference exceeding 5 bpm or 10%; for respiratory rate, as a difference greater than 3 breaths per minute or 10% [18]. Within the GEE analysis, an exchangeable correlation structure was applied, followed by a backward selection procedure including clinically relevant covariates such as heart rate, respiratory rate, blood pressure, heart rhythm category, selected laboratory values, and the use of continuous intravenous medication. An overview of all candidate predictors is provided in Appendix I. Notably, no imputation was performed as missing values in vital sign parameters could not be reliably inferred from other clinical data, and because these parameters are considered clinically independent. The Fitzpatrick skin tone variable (a six-point scale ranging from very fair [Type I] to very dark [Type VI]) was not included in the analysis due to lack of variability, as all patients had skin types I–II. Hence, a complete case analysis was conducted. The final models were evaluated using both discrimination and calibration metrics.

Data analysis was performed using RStudio (version 2023.06.0).

## Results

### Population

**Fig. 2:**
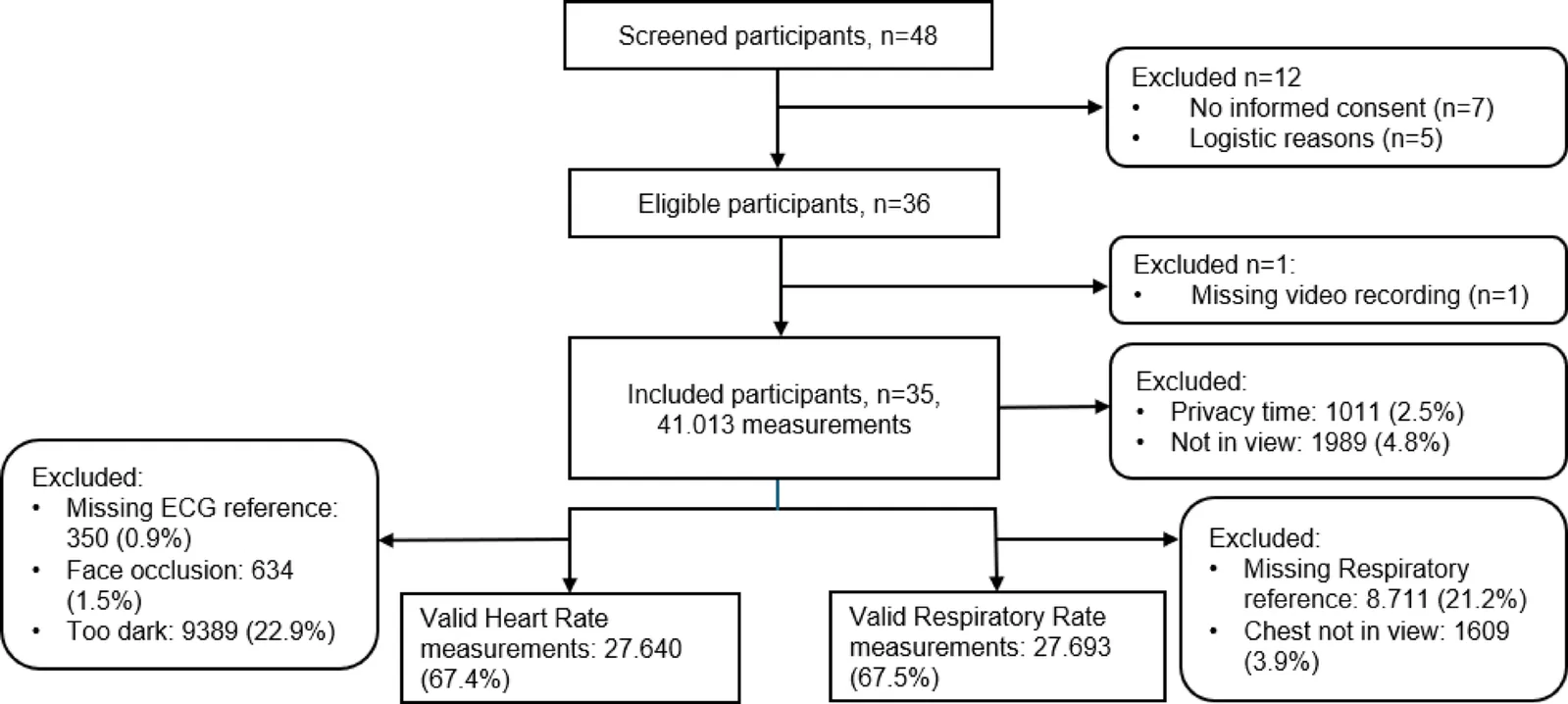
Study flowchart (1 measurement = 60-second interval with heart/respiratory rate and corresponding reference value)

Out of a screened total of 48 patients, 35 patients were included in the study (Fig. 2; Table 1). Seven patients declined participation, five could not be scheduled in the designated study room, and one was excluded due to missing video data caused by a recording failure. The total recording duration was 683.6 h, with an average per patient of 19.5 h (SD 7.3). Of the total recording duration, 67.4% of heart rate and 67.5% of respiratory rate were valid recording time (Fig. 2).

**Table 1:** Patient characteristics (IQR: interquartile range)

Patient characteristics (*N* = 35)		
Sex, n (%)	Male Female	22 (63) 13 (37)
Age, yr, median (IQR)		69 (13)
BMI, kg/m^2^, median (IQR)		28 (4)
Skin type, n. (%)	Fitzpatrick I-II Fitzpatrick III-VI	35 (100) 0
Admission reason ICU, n (%)	Elective cardiac surgery Coronary artery bypass grafting (CABG) Aortic valve replacement (AVR) Mitral valve replacement (MVR) Combined CABG/AVR Supra coronary ascendens replacement (SCAR) Combined valve replacement Non-elective admission Respiratory insufficiency Shock	24 (69) 8 (28) 4 (11) 2 (6) 4 (11) 4 (11) 2 (6) 4 (11) 7 (20)

### Measurement error and reliability

**Fig. 3:**
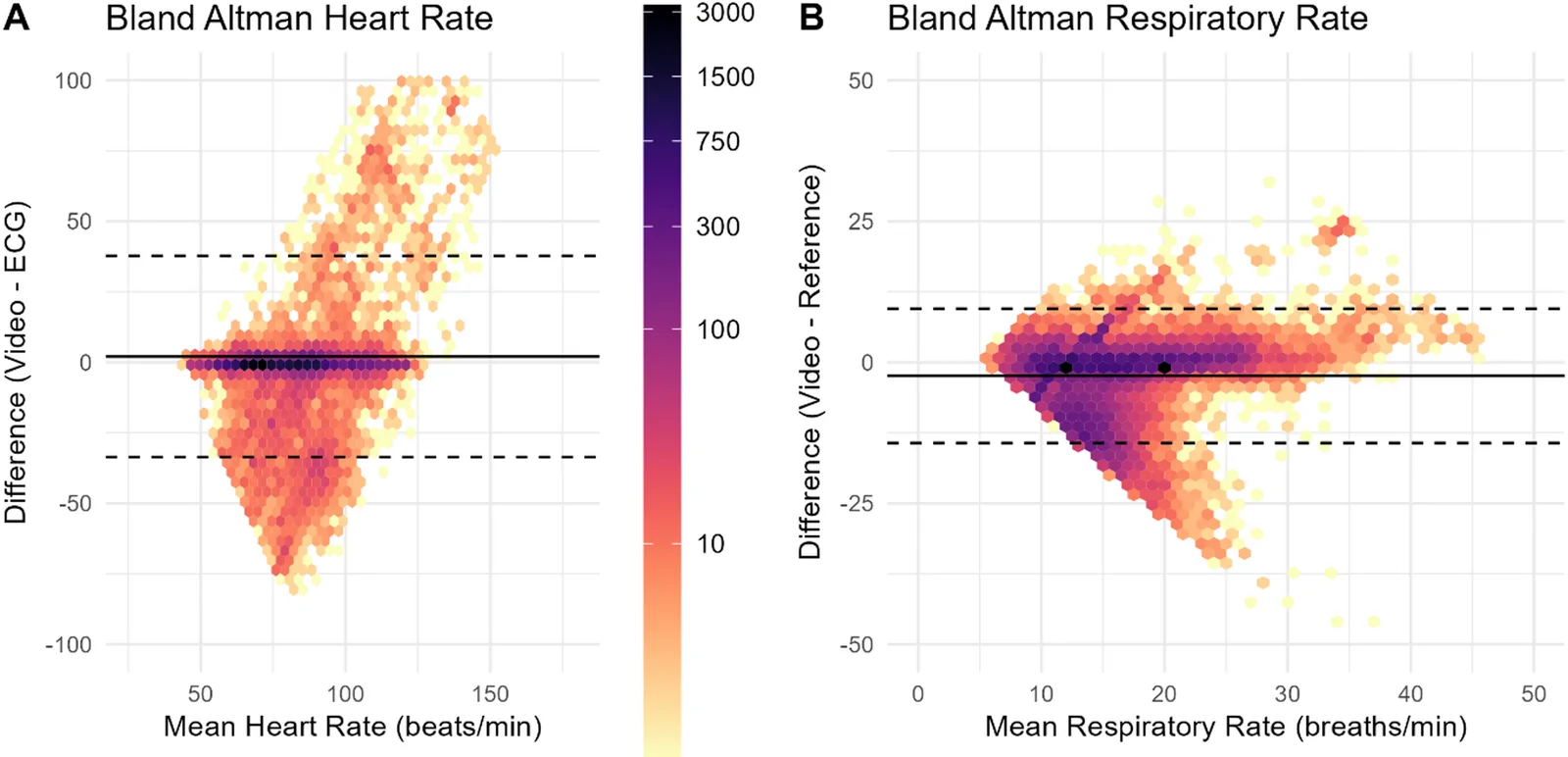
Agreement between the video-based **(A)** heart rate and **(B)** respiratory rate and reference measurements is visualized by plotting the difference between both signals on the y-axis against the mean heart rate or respiratory rate on the x-axis. Each data point represents a 60-second interval. The solid black line indicates the bias, while the dotted lines represent the limits of agreement

For heart rate, the mean bias was 2.1 beats per minute (bpm), with LoA ranging from − 33.6 to 37.7 bpm (Table 2). For respiratory rate, the mean bias was − 2.2 breaths per minute, with LoA from − 15.1 to 10.6 breaths per minute (Table 2). As illustrated in Fig. 3A and B, the interpretation of these agreement estimates requires caution, as heteroscedasticity for heart rate and proportional bias for respiratory rate indicate that measurement error varied across the measurement ranges. Although the LoA were relatively wide for both parameters, the majority of measurements fell within clinically accepted accuracy thresholds: 81.9% for heart rate (deviations < 5 bpm) and 63.5% for respiratory rate (deviations < 3 breaths per minute). Larger deviations were predominantly observed at higher heart rate. The intraclass correlation coefficients were 0.43 for heart rate and 0.41 for respiratory rate, indicating moderate agreement and reflecting variability in measurement accuracy across the respective ranges. A subgroup analysis evaluating respiratory rate accuracy in mechanically ventilated versus non-ventilated patients has been added to Appendix III.

**Table 2:** Measurement error and reliability of video technology compared with the reference devices. SEs and 95% CIs around the LoA were calculated to account for the repeated measurements within a patient, incorporating within- and between-patient variability and correcting for unequal observations per patient (harmonic mean). *(BPM: beats per minute*,* BRPM: breaths per minute*,* CI: confidence interval*,* ICC: intraclass correlation*,* LLOA: lower limit of agreement*,* SE: standard error*,* ULoA: upper limit of agreement)*

Mean (range)	HR	RR
	81 (42–142)	16 (0–60)
Bland Altman Bias (bpm/ brpm, SE) LLoA (bpm/brpm, 95% CI) ULoA (bpm/ brpm, (95% CI) Within 5 bpm/3 brpm (n, %) Within 10% (n, %)	2.1 (1.3) -33.6 (-37.0/-30.7) 37.7 (34.9/41.1) 22 643 (81.9) 23 343 (84.5)	-2.4 (0.4) -14.3 (-15.1/-13.6) 9.5 (8.8/10.3) 17 591 (63.5) 14 077 (50.8)
ICC	0.43 (0.30/0.53)	0.41 (0.28/0.51)
r	0.53 (0.52/0.54)	0.47 (0.46/0.48)

### Clinical accuracy

**Fig. 4:**
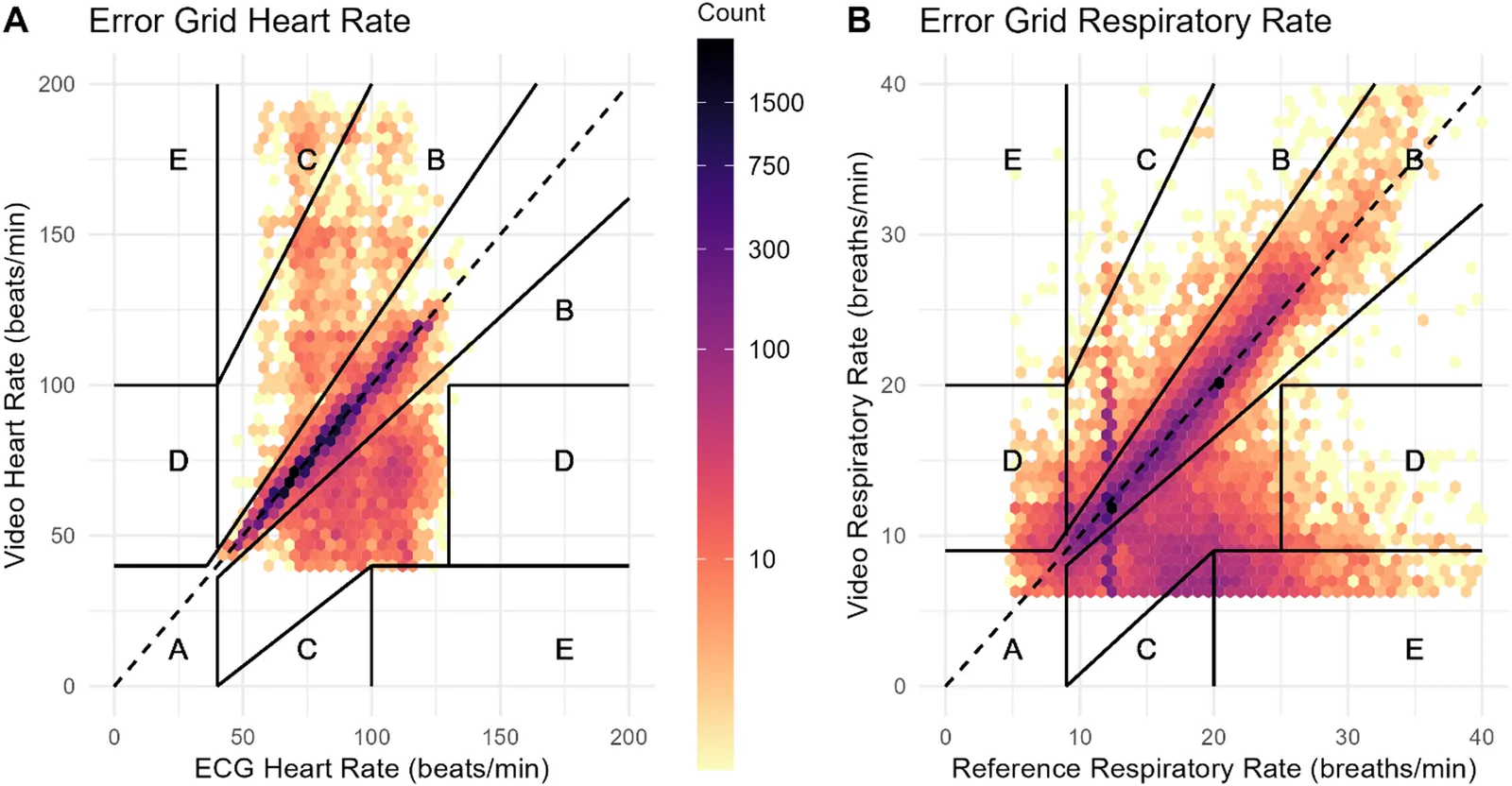
Clarke error grid analysis, with each colored dot representing a 60-second interval. **A**. Heart rate **B**. Respiratory rate

Error grid analysis results show that for heart rate, most measurements fall within Zone A, with the majority of the remaining data points in Zone B (Table 3; Fig. 4). For respiratory rate, the majority of measurements (87.5%) are in Zones A and B, while 12.5% fall within Zones C, D, or E (Table 3; Fig. 4).

**Table 3:** Distribution of heart rate (HR) and respiratory rate (RR) measurements across error grid zones. Values are presented as counts with percentages in parentheses, indicating the proportion of measurements falling within each zone

	HR	RR
A B C D E	24 227 (87.7) 3212 (11.6) 200 (0.7) 1 (0.0) 0 (0.0)	17 672 (63.8) 6572 (23.7) 881 (3.2) 945 (3.4) 1623 (5.9)

### Influence of clinical factors on agreement

In the GEE analysis of heart rate discrepancy, higher heart rate, the presence of atrial fibrillation, norepinephrine administration, and patient movement were all independently associated with poorer agreement between video-based and reference measurements (Table 4). For respiratory rate, patient movement was the only significant predictor of discrepancy (Table 5). As patient movement was the only significant predictor of discrepancy in respiratory rate, no multivariable model was fitted for this outcome. Respiratory rate level was not a significant predictor, indicating that measurement accuracy was maintained across the full respiratory rate range. These associations remained statistically significant in the multivariable model, although the effect sizes for heart rate and atrial fibrillation were attenuated (Table 6). An overview of the occurrence of all candidate predictors is provided in Appendix II.

**Table 4:** Univariable analysis heart rate model (AF: atrial fibrillation, CI: confidence interval, OR: odds ratio, SR: sinus rhythm)

Predictors	OR	95% CI	*p*-value
*Vital signs* Heart rate > 100 bpm Heart rhythm: SR vs. AF SR vs. other	2.2 3.2 1.2	1.2–4.0 2.2–4.7 0.9–1.4	0.008 < 0.001 0.07
*Medication (continuous iv)* Noradrenaline 100mcg/ml: 0 vs. 0-10 ml/h 0 vs. > 10 ml/h	1.5 2.5	1.0–2.3 1.5–3.4	0.05 < 0.001
Movement No vs. Slight movement No vs. Substantial movement	1.5 2.5	1.1 – 2.1 1.8–3.3	0.008 < 0.001

**Table 5:** Univariable analysis respiratory rate model (CI: confidence interval, OR: odds ratio)

Covariates	OR	95% CI	*p*-Value
Movement No vs. Slight movement No vs. Substantial movement	3.5 7.8	2.8–4.5 5.6–10.7	< 0.001 < 0.001

**Table 6:** Multivariable analysis heart rate model (AF: atrial fibrillation, CI: confidence interval, OR: odds ratio, SR: sinus rhythm

Covariates	OR	95% CI	*p*-value
*Vital signs* Heart rate > 100 bpm Heart rhythm: SR vs. AF SR vs. other	1.8 2.2 1.1	1.1–3.0 1.5–3.4 0.9–1.2	0.02 < 0.001 0.4
*Medication (continuous iv)* Norepinephrine 100mcg/ml: 0 vs. 0-10 ml/h 0 vs. > 10 ml/h	1.9 2.6	1.3–2.7 1.5–4.5	< 0.001 < 0.001
Movement No vs. Slight movement No vs. Substantial movement	1.7 2.8	1.2 – 2.2 2.2–3.6	< 0.001 < 0.001

**Fig. 5:**
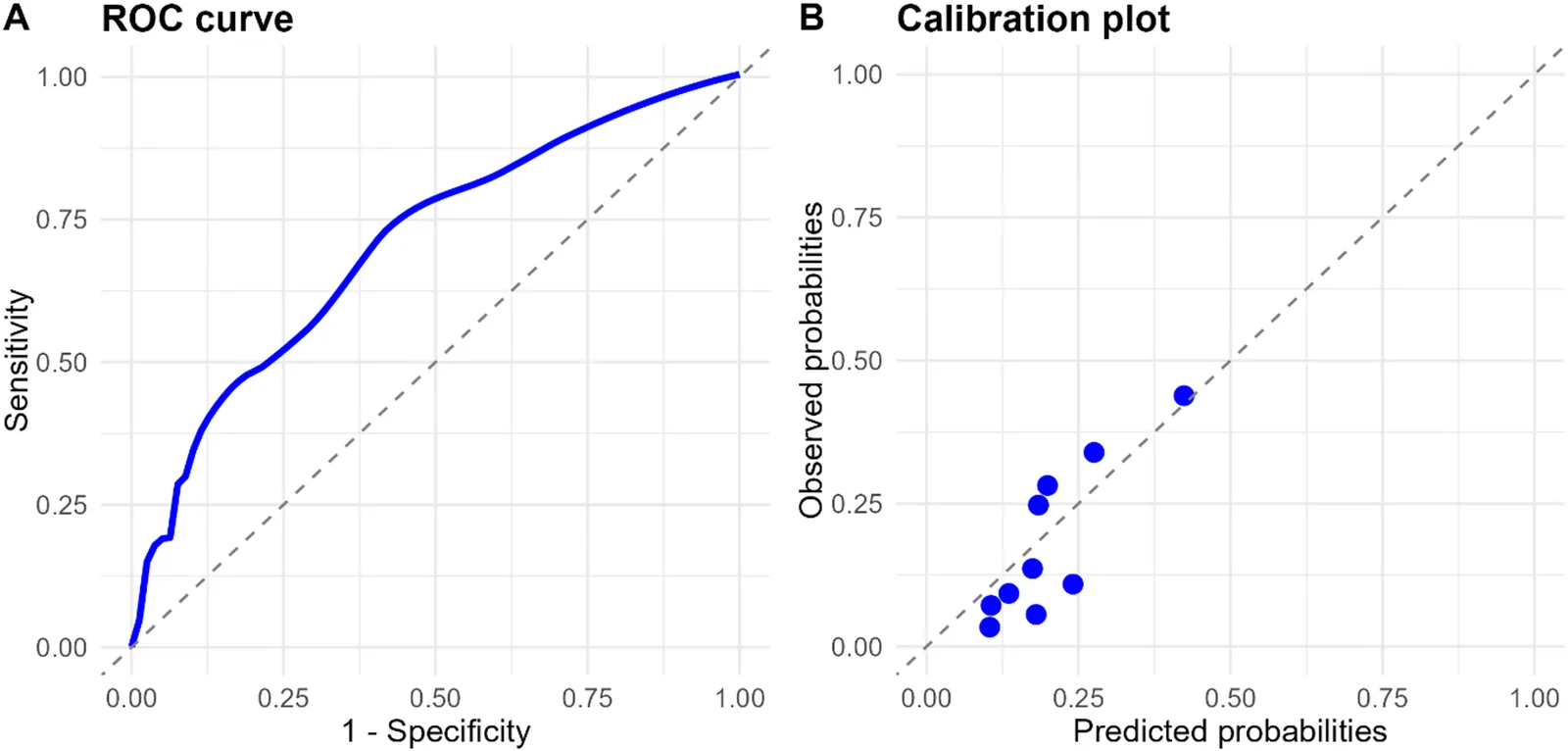
**(A)** ROC curve heart rate model **(B)** Calibration plot heart rate model

**Fig. 6:**
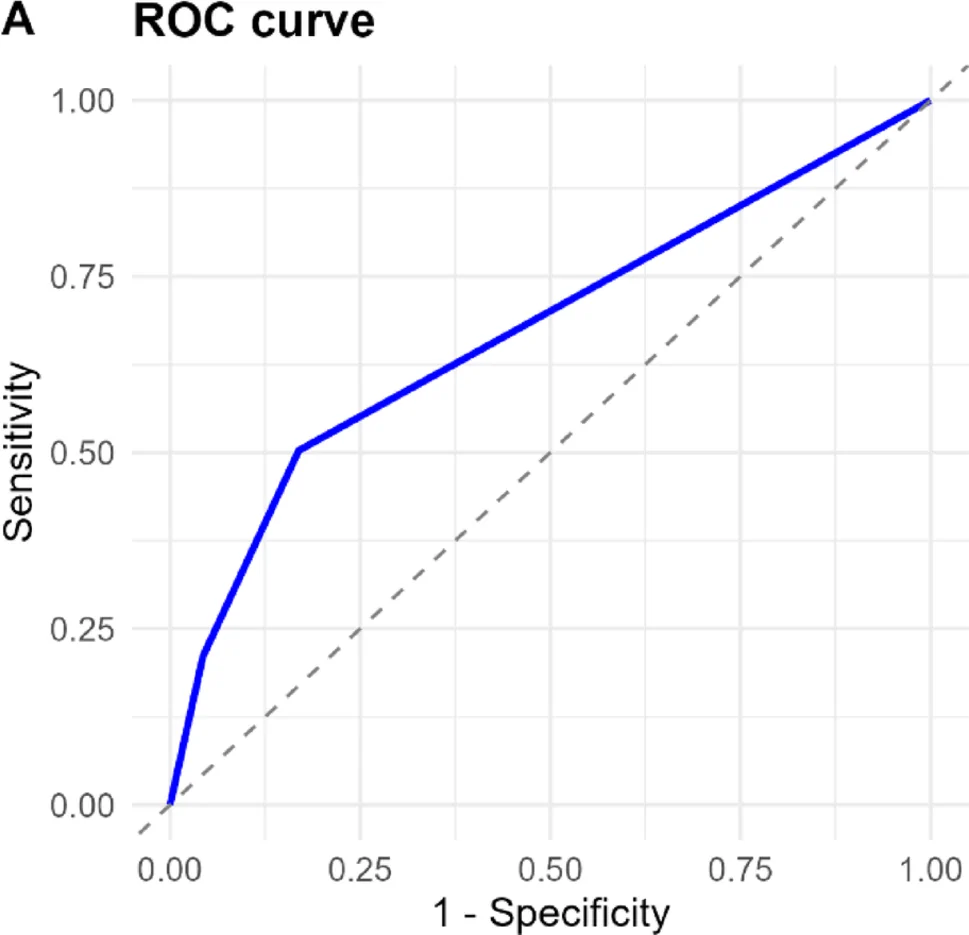
ROC curve respiratory rate model

The ROC curves (Figs. 5a and 6) showed an area under the curve (AUC) of 0.71 (0.70–0.72) for heart rate and 0.67 (0.66–0.68) for respiratory rate, which is indicative of moderate discriminative ability. Calibration was assessed using calibration plots (Fig. 5b). For the heart rate model, predicted probabilities were mostly concentrated in the lower range, reflecting the low prevalence of the outcome. This distribution may have limited the model’s ability to identify higher-risk individuals and thereby affected overall calibration. For the respiratory rate model, which was based on a single categorical predictor with three levels, a calibration plot was considered uninformative and therefore not presented.

## Discussion

This prospective ICU validation study evaluated video-based monitoring of heart rate and respiratory rate in a high-acuity setting, serving as an evaluation setting to assess robustness across a broad spectrum of pathophysiological conditions. Heart rate measurements showed strong agreement with reference values, with only modest performance reductions during several physiological states. Respiratory rate estimation was more affected by motion, resulting in lower accuracy; however, clinically relevant abnormalities were consistently detected. By leveraging the heterogeneity and high incidence of abnormal vital signs in critically ill patients, this study provides insight into measurement robustness and potential failure modes, informing implementation in lower-acuity care settings.

Most previous studies evaluating video-based vital signs have been conducted under controlled conditions, typically involving short monitoring durations or healthy volunteers [1, 2]. Only a few have investigated video technology performance over extended periods in clinical settings but in relatively stable patients.

Notably, our heart rate results demonstrate better coverage than those reported in previous studies [3, 19], whereas our respiratory rate coverage is comparable [3, 19]. Previous work on heart rate in 15 ICU patients displayed a bias and LoA of − 2.0 [-6.4;2.5] beats per minute, and − 1.4 [-6.6;3.9] for respiratory rate [3]. Although these agreement metrics appear more favorable than those observed in our study, it is important to note that they applied a signal quality filter to exclude low-confidence data. While this approach improved performance estimates, it also resulted in the exclusion of 46.8% of the heart rate data and 36.9% of the respiratory rate data, making direct comparison challenging.

Consistent with prior work, our study showed that agreement between video-derived and reference measurements for both heart rate and respiratory rate decreased significantly during periods of patient motion [20]. This finding highlights the continued challenge of ensuring signal robustness in real-world clinical environments. Moreover, we observed lower agreement during cardiac arrhythmias and at higher heart rates [10]. In contrast to a prior study, reduced agreement during norepinephrine use was found, whereas that study reported worse agreement during episodes of low blood pressure, which we did not observe [21]. It must be noted that their analysis was based on a substantially smaller sample size (35 h) and used signal-to-noise ratio as the outcome measure, rather than clinical agreement metrics. These methodological differences may partly explain the discrepancies and also raise questions about the clinical relevance and generalizability of the findings.

Regarding effective trend monitoring on general hospital wards, it is essential to understand under which conditions video-based vital sign monitoring performs reliably. Interestingly, our findings indicate that the technology is less accurate during episodes of elevated heart rate and in the presence of cardiac arrhythmias. However, the error grid analysis demonstrated a clinical accuracy of 99.3%, suggesting that the clinical relevance of these errors is limited. The reduced accuracy is likely explained by the reduction in pulse amplitude that occurs both during arrhythmias and at higher heart rates, which may compromise signal quality. This raises the possibility that such deviations could be used as a signal in themselves rather than solely being limitations, as contactless monitoring may help differentiate between stable patients and those who require additional medical attention. Algorithms that detect these patterns could support timely escalation of care when needed.

In addition, the use of vasopressors such as norepinephrine was associated with decreased agreement, likely due to peripheral vasoconstriction reducing the video-based heart rate signal. Importantly, vasoconstriction is not limited to pharmacological causes but may also occur as part of the physiological response to clinical deterioration. As a result, changes in peripheral perfusion in deteriorating patients could similarly influence signal quality and measurement accuracy, which should be considered when evaluating video-based monitoring technologies. Moreover, video data contain valuable contextual information, enabling advanced algorithms to compensate for environmental influences and potentially detect other clinically relevant changes in patient condition.

Respiratory rate estimation using video-based technology was particularly sensitive to patient movement. The latter was the only factor in our study that consistently predicted lower agreement between video-derived and reference respiratory rate values. However, this limitation is not unique to video technology. Conventional methods such as impedance pneumography and capnography and wearables devices are also known to produce unreliable measurements during motion or activities such as talking [5, 22].

Finally, several clinical factors (e.g. blood pressure, Spo2) did not influence the accuracy of video-based heart rate and respiratory rate measurements, underscoring robustness across different clinical contexts and supporting potential applicability in heterogeneous ward populations. Nevertheless, overall model discrimination was moderate (AUC 0.71 for heart rate and 0.67 for respiratory rate), indicating that additional determinants of measurement agreement remain to be identified.

This study has several limitations. First, as participants were recruited in Eindhoven, the Netherlands, the study population predominantly consisted of individuals with lighter skin tones. Therefore, the generalizability of our findings to individuals with darker skin tones may be limited. Prior research indicates that video signal quality can be affected by skin pigmentation due to differences in light absorption and reflection, which may reduce performance in these population [10]. Evaluating the performance of video-based monitoring across diverse populations is therefore important to ensure accurate and equitable clinical implementation.

Second, we used 60-second measurement windows for heart rate and respiratory rate in this study. This choice was primarily driven by our aim to investigate the influence of clinical factors, such as hypotension and low oxygen saturation, on video monitoring performance. Using substantially longer windows (e.g., five minutes) would risk averaging out episodes in which these disturbances occur, thereby obscuring their effects, especially in the ICU where such conditions are typically identified and treated promptly. At the same time, this approach aligns with the intended clinical application on general hospital wards, where the goal is to monitor trends over time rather than capture beat-to-beat variability. Shorter windows could make the system more responsive to acute changes, but would also increase susceptibility to transient signal artifacts. A 60-second median therefore offered a practical balance between clinical relevance and signal stability.

Another limitation of this study is that a substantial proportion of the collected data could not be included in the final analysis, with approximately 67% of heart rate and respiratory rate measurements meeting the inclusion criteria. This was partly due to the reliance of the RGB-based system on sufficient ambient lighting, as some recordings had to be excluded because of insufficient light conditions. This highlights the dependency of RGB-based monitoring on environmental lighting. To address this limitation, ongoing work is focused on integrating near-infrared (NIR) cameras, which enable monitoring independent of visible light conditions and may improve the feasibility of continuous monitoring in low-light environments. However, evaluating the combined use of RGB and NIR signals was beyond the scope of the present study.

Moreover, the ICC values and Bland–Altman limits of agreement should be interpreted with caution, as the analysis was performed on the full dataset without applying a signal quality filter. Consequently, measurements affected by motion artefacts or poor signal quality were retained, resulting in several outliers that disproportionately influenced the agreement metrics and widened the limits of agreement (− 33.6 to 37.7 bpm), thereby potentially underestimating the reliability of the method under conditions of adequate signal quality. This also explains the apparent discrepancy between the relatively wide limits of agreement and the high proportion of heart rate measurements falling within clinically acceptable zones A and B (99.3%) in the Error Grid analysis. While Bland–Altman analysis is sensitive to extreme deviations, the Error Grid reflects clinical impact, demonstrating that most discrepancies did not affect clinical decision-making.

Lastly, the reference method used for respiratory rate measurement, impedance pneumography, is known to have limitations in accuracy, particularly during patient movement or irregular breathing patterns [23]. Variability in signal quality, electrode placement, and non-respiratory changes in thoracic impedance may introduce measurement error. In spontaneously breathing patients, respiratory rate was derived from impedance pneumography, using the values provided by the clinical monitor, representing the processed respiratory rate estimates routinely used in clinical practice. In ventilated patients, respiratory rate was derived from the ventilator flow signal, which provides a more reliable reference. Consequently, part of the disagreement between video-based and reference respiratory rates may reflect imperfections in the reference method rather than deficiencies of the video approach. Nonetheless, impedance pneumography is the clinical standard for continuous monitoring in spontaneously breathing patients and was therefore considered an appropriate comparator. The absence of a universally accepted continuous reference standard for respiratory rate in non-ventilated patients remains a known challenge in validation studies of respiratory monitoring technologies.

Future research should focus on integrating the identified predictors into a quality metric that distinguishes between high- and low-quality signals in clinical practice. Such a metric could support real-time assessment of data reliability. Validation of this approach in other clinical settings, such as the general ward, is needed to assess its robustness beyond the ICU. Incorporating additional vital signs and contextual factors may further enhance performance. Ultimately, prospective studies would be required to evaluate whether these methodological improvements translate into clinically meaningful benefits.

## Conclusions

Video-based heart rate measurements are largely accurate and clinically reliable, video-based respiratory rate estimation requires further improvements, particularly under motion. Continued optimization of signal processing and clinical integration strategies will be crucial for enabling safe and effective implementation in routine care.

## Supplementary Information

Below is the link to the electronic supplementary material.

Supplementary Material 1

## Data Availability

The datasets generated and analyzed during this study are not publicly available due to their sensitive and identifiable nature, as well as the restrictions imposed by the ethics protocol to safeguard the privacy of the patients involved.
