# Supplementary material for: Clinical validation of video-based vital sign monitoring in the intensive care unit: a prospective cohort study: Clinical validation of video-based vital sign monitoring
Source: J Clin Monit Comput. 2026 May 13;40(5):1113–23. doi: 10.1007/s10877-026-01440-0 (PMC13562297; doi:10.1007/s10877-026-01440-0)
Supplement: Supplementary file 1 [file 10877_2026_1440_MOESM1_ESM.docx]

**Appendix I**. List of candidate predictors of GEE analysis

| Predictors | Category |
| --- | --- |
| *Vital signs*  Heart rate (<100bpm, > 100bpm)  Heart rhythm (Sinus rhythm, atrial fibrillation, other)  Respiratory rate (<12, 12-20, >20)  Spo2 (<92, >92)  Blood pressure (MAP <65, >65) | Dichotome  Categorical  Categorical  Dichotome  Dichotome |
| *Medication (continuous iv)*  Norepinephrine 100mcg/ml (0 ml/h, 0-10ml/h, >10ml/h)  Milrinone (yes/no)  Dobutamine (yes/no)  Amiodarone (yes/no)  Propofol (yes/no)  Nicardipine (yes/no)  Dexmedetomidine (yes/no) | Categorical  Dichotome  Dichotome  Dichotome  Dichotome  Dichotome  Dichotome |
| Movement  No movement, Slight movement, Substantial movement | Categorical |

**Appendix II.** Occurrence of candidate predictors

| Predictors | Amount | Missings |
| --- | --- | --- |
| *Vital signs*  Heart rate  < 100bpm)  > 100 bpm  Heart rhythm  Sinus rhythm  Atrial fibrillation  other  Respiratory rate  <12,  12-20  >20  Spo2  <92%  >92%  Blood pressure  MAP <65  MAP > 65 | 33.068  5.708  18.675  3.274  16.152  10.006  13.508  9851  2500  35.418  6.508  29.192 | 769  1.444  6180  1.627  3.845 |
| *Medication (continuous iv)*  Norepinephrine 100mcg/ml  0 ml/h  0-10ml/h  >10ml/h  Milrinone  yes  no  Dobutamine  yes  no  Amiodarone  yes  no  Propofol  yes  no  Nicardipine  yes  no  Dexmedetomidine  yes  no | 23.141  12.635  3.769  4.776  34.769  659  38.344  1.337  38.208  7.410  32.135  2.191  37.354  661  38.884 | 0  0  0  0  0  0  0 |
| Movement  No movement  Slight movement  Substantial movement | 3.925  28.378  7.241 | 0 |

**Appendix III.** Subgroup analysis of respiration agreement with and without mechanical ventilation

|  | RR_ventilation | RR_without ventilation |
| --- | --- | --- |
| Bland Altman  Bias (bpm/ brpm, SE)  LLoA (bpm/brpm, 95% CI)  ULoA (bpm/ brpm, (95% CI)  Within 3 brpm (n, %) | -2.6 (1.7)  -14.3 (-19.4/-11.7))  9.1 (6.5/14.3)  3923 (83.1) | -2.2 (0.4)  -15.4 (-16.5/-14.6))  10.9 (10.0/11.9))  13690 (58.2) |
